# Mechanistic Effects of Aerobic Exercise in Alzheimer's Disease: Imaging Findings From the Pilot FIT-AD Trial

**DOI:** 10.3389/fnagi.2021.703691

**Published:** 2021-10-07

**Authors:** Fang Yu, Michelle A. Mathiason, SeungYong Han, Jeffrey L. Gunter, David Jones, Hugo Botha, Clifford Jack

**Affiliations:** ^1^Arizona State University Edson College of Nursing and Health Innovation, Phoenix, AZ, United States; ^2^University of Minnesota School of Nursing, Minneapolis, MN, United States; ^3^Mayo Clinic Department of Radiology, Rochester, MN, United States

**Keywords:** exercise, Alzheimer's disease, dementia, imaging, MRI, hippocampal volume, white matter hyperintensity

## Abstract

Despite strong evidence from animal models of Alzheimer's disease (AD) supporting aerobic exercise as a disease-modifying treatment for AD, human mechanistic studies are limited with mixed findings. The objective of this pilot randomized controlled trial was to examine the effects of 6-month aerobic exercise on hippocampal volume, temporal meta-regions of interest (ROI) cortical thickness, white matter hyperintensity (WMH) volume, and network failure quotient (NFQ), measured with MRI, in community-dwelling older adults with AD dementia. Additionally, the relationships between 6- and 12-month changes in MRI biomarkers and the AD Assessment Scale-Cognition (ADAS-Cog) were examined. Sixty participants were randomized, but one was excluded because baseline MRI failed quality control: 38 randomized to cycling and 21 to stretching. The intervention was moderate-intensity cycling for 20–50 mins, three times a week for 6 months. Control was low-intensity stretching. The study outcomes include hippocampal volume, temporal meta-ROI cortical thickness, WMH volume, and NFQ. Outcomes were measured at baseline, 6 months, and 12 months. The sample averaged 77.3 ± 6.3 years old with 15.6 ± 2.9 years of education and 53% men. Both groups experienced significant declines over 6 months in hippocampal volume (2.64% in cycling vs. 2.89% in stretching) and temporal meta-ROI cortical thickness (0.94 vs. 1.54%), and over 12 months in hippocampal volume (4.47 vs. 3.84%) and temporal meta-ROI cortical thickness (2.27 vs. 1.79%). These declines did not differ between groups. WMH volume increased significantly with the cycling group increasing less (10.9%) than stretching (24.5%) over 6 months (*f* = 4.47, *p* = 0.04) and over 12 months (12.1 vs. 27.6%, *f* = 5.88, *p* = 0.02). NFQ did not change significantly over time. Pairwise correlational analyses showed a significant negative correlation between 6-month changes in hippocampal volume and ADAS-Cog (*r* = −0.34, *p* < 0.05). To conclude, aerobic exercise may reduce the decline in hippocampal volume and temporal meta-ROI cortical thickness during the intervention period, but the effect sizes are likely to be very small and dose-dependent and reverse once the intervention stops. Aerobic exercise is effective on slowing down WMH progression but has no effect on NFQ. Hippocampal atrophy was associated with cognitive decline during the intervention period.

**Clinical Trial Registration:** www.ClinicalTrials.gov, identifier: NCT01954550.

## Introduction

Dementia affects 47 million people worldwide and is projected to afflict 150 million by 2050 (International, 2016). Alzheimer's disease (AD) is the most common cause of dementia, accounting for 60–80% of all dementia cases (Alzheimer's Association, 2021). No drugs can yet prevent, slow down, or cure AD, and the current Food and Drug Administration (FDA)-approved treatments don't affect AD pathology (Matsunaga et al., 2019). Even when targeting AD neuropathology of abnormal amyloid-beta (Aβ) and tau [hyperphosphorylated tau (p-tau)], recent promising drug trials continued to fail (Tolar et al., 2020). Reasons for these failures are myriad and likely attributable to the facts that these treatments did not address the multifactorial nature of AD neuropathogenesis; the dominant amyloid hypothesis has limited the investigations of other causative factors of AD; and reliance on AD animal models for treatment discovery overly simplifies the complex nature of human cognition, behaviors, emotions, and disease chronicity (Banik et al., 2015). Hence, mechanistic studies on and beyond Aβ and tau biomarkers in humans are critical for developing disease-modifying treatments in AD.

Over the past two decades, aerobic exercise has emerged as a potential disease-modifying treatment for AD. In AD-transgenic animal models, aerobic exercise has been shown to favorably modify the accumulation, degradation, and removal of Aβ and p-tau as well as other abnormal processes occurring in AD such as neuroinflammation (McGurran et al., 2019; da Costa Daniele et al., 2020). On the molecular level, aerobic exercise was found to stimulate the production and function of brain-derived neurotrophic factors (BDNF). BDNF contributes to neurogenesis (particularly in hippocampi), neuronal survival, and synaptic plasticity and mediates memory improvement (Cotman and Berchtold, 2007). Mechanistic studies of aerobic exercise in humans are limited with mixed findings. Observational studies showed that physical activity or exercise was positively (Frederiksen et al., 2019a; Raichlen et al., 2019), not (Best et al., 2015), or negatively (Wagner et al., 2015) associated with hippocampal volume in cognitively normal adults. Self-reported high-intensity physical activity was associated with lower tau in the cerebrospinal fluid (CSF) among cognitively normal older adults (Baker et al., 2012). Engagement in moderate, but not light or vigorous, physical activity was associated with higher CSF Aβ42 and lower CSF total tau and p-tau in asymptomatic late-middle-aged adults at risk for AD (Law et al., 2018). High level of self-reported physical activity was also associated with lower levels of plasma Aβ (Brown et al., 2013), Positron Emission Tomography-qualified Aβ (Liang et al., 2010; Head et al., 2012; Okonkwo et al., 2014), and PET-quantified tau (Brown et al., 2018) in cognitively intact older adults. In contrast, other studies found no associations between physical activity and PET-quantified in this population (de Souto Barreto et al., 2015) or CSF Aβ (Brown et al., 2017). However, self-reported measures of physical activity were prone to recall errors and biases due to varied interpretations of physical activity levels.

Randomized controlled trials (RCTs) to establish the disease-modifying effects of aerobic exercise are even more limited than observational studies. Using MRI in cognitively normal older adults, some studies reported that aerobic exercise increased prefrontal lobe volume (Tamura et al., 2015), gray and white matter volumes in the anterior cingulate (Colcombe et al., 2006), and hippocampal volume (Erickson et al., 2011; Niemann et al., 2014), but other studies showed no (Best et al., 2015) or detrimental effects on brain and hippocampal volume (Wagner et al., 2015). The latter study, however, was conducted with young men only, had large between-participant variations in hippocampal volume changes, and was a short intervention of 6 weeks (Wagner et al., 2015). In older women with mild cognitive impairment (MCI), 6-month aerobic exercise was found to significantly increase hippocampal volume (ten Brinke et al., 2015). In older adults with MCI or mild AD dementia, 26-week aerobic exercise reduced hippocampal atrophy (hippocampal volume in the intervention group decreased 0.8% vs. 1.6% in the control group) (Morris et al., 2017). In older adults with mild-to-moderate AD dementia, 16-week aerobic exercise had no effects on hippocampal volume (Frederiksen et al., 2018), CSF Aβ (Jensen et al., 2016), PET-quantified Aβ (Frederiksen et al., 2019b), and CSF tau (Jensen et al., 2017). Furthermore, emerging findings suggest that aerobic exercise may improve cortical thickness (Bae et al., 2020), functional connectivity (Boa Sorte Silva et al., 2020), and white matter hyperintensity (WMH) (Graff-Radford et al., 2019). Together, these findings suggest the need to examine whether and how aerobic exercise may modify limbic neurodegeneration in humans.

The objective of this pilot RCT, the FIT-AD Trial, was to examine the effects of 6-month aerobic exercise on hippocampal volume, temporal meta-regions of interest (ROI) cortical thickness, WMH volume, and network failure quotient (NFQ) in community-dwelling older adults with mild-to-moderate AD dementia. FIT-AD stands for Functional Impact of aerobic exercise Training in Alzheimer's Disease. We hypothesized that intervention participants will have a smaller decrease in hippocampal volume, cortical thickness, and NFQ and a smaller increase in WMH volume over 6 and 12 months in comparison to stretching controls. Further, we examined the correlations of the longitudinal changes of these MRI biomarkers and cognition over 6 and 12 months.

## Materials and Methods

### Design

The FIT-AD Trial was a pilot RCT that followed the CONSORT guideline with its CONSORT checklist (Eldridge et al., 2016) provided in the Supplementary Table. It first qualified participants based on their eligibility for participating in the exercise interventions. Those who met the eligibility criteria were then approached for their interest in volunteering for the MRI component of the trial, and, if interested, assessed for MRI eligibility. Randomization was performed at the main study level (not the MRI eligibility level) to allocate participants to 6-month intervention (moderate-intensity cycling) or control (low-intensity stretching exercise) for 20–50 mins per session, three times a week on a 2:1 ratio with three age strata (66–75, 76–85, and 85+ years of age). Allocation was generated and concealed to all data collectors and investigators except for the biostatistician. MRI was completed at baseline before randomization and at 6 and 12 months. The biostatistician generated the randomization sequence that was sequentially concealed in an opaque envelop. Once a participant was enrolled, the study interventionist opened the envelop to reveal the group assignment of a participant. This trial was approved by the University of Minnesota's Institutional Review Board (IRB: #1306M35661). The detailed study protocol was published previously (Yu et al., 2014).

### Setting

MRI was conducted using 3 Tesla (3T) Siemens Trio system (Siemens, Erlangen, Germany) at the university Center for Magnetic Research and Resources. The MRI protocol was set up and qualified on site by our MRI team located at the Mayo Clinic Aging and Dementia Imaging Research (ADIR) Lab. All scans were securely transmitted to the ADIR lab for evaluation of protocol compliance, scan quality, medical abnormality, and study eligibility. All MRI personnel were blinded to participant group assignment. Any issues were communicated and resolved accordingly. Exercises were delivered in a Young Men's Christian Association gym or the lounge of a senior community.

### Participants

Participants were first qualified for the FIT-AD Trial. Community-dwelling older adults, who had a clinical diagnosis of AD dementia, were 66 years old and older, and spoke English were potentially eligible if they scored 15–26 on the Mini-Mental State Examination (MMSE) and 0.5–2 on Clinical Dementia Rating (CDR), had medical clearance for exercise and MRI, and were stable on AD drugs >1 month if prescribed. Potential participants were excluded if their resting heart rate was ≤ 50 or ≥100 beats per minute, had neurologic, psychiatric disorders, alcohol/chemical dependency that explained their dementia, exercise contraindications, new symptoms or diseases that had not been evaluated by their providers, and abnormal findings from the symptom-limited cycler-ergometer test. This study was powered on the primary cognitive outcome, not the MRI outcomes (Yu et al., 2014).

The inclusion criteria for the MRI component included consent to volunteer for the MRI and passed MRI safety screening. Participants were excluded from both the MRI component and the main study if MRI showed abnormality (normal pressure hydrocephalus, brain tumor, subdural hematoma, significant posttraumatic encephalomalacia, or one or more large hemispheric infarctions).

A variety of strategies were used for recruitment such as Alzheimer's Association's events, referrals, and flyer/brochure distributions. Recruitment started in March 2014 and ended in March 2019, and the last follow-up was completed in October 2019. Participants were screened through (1) phone screen; (2) in-person interview (consent, MMSE, CDR); (3) medical clearance (exercise/MRI safety); and (4) symptom-limited peak cycle-ergometer test (unknown heart conditions) and MRI if qualified. After completing baseline data collection, participants were enrolled and started their assigned exercise within a week (Yu et al., 2014).

### Intervention

The target intervention was supervised, individualized, moderate-intensity cycling on recumbent stationary cycles for 50 mins a session, three times a week, after an adjustment period.

Moderate intensity was prescribed as 65–75% of heart rate reserve (HRR) and 12–14 on the 6–20 Borg Ratings of Perceived Exertion (RPE) scale. The adjustment period was essential to safely progress participants to the target intensity and session duration over time. Hence, the intervention sessions started at a lower than the target dose (50–55% of HRR or RPE 9–11 for 20 mins) in the first week. The session intensity and duration were alternately increased by 5% of HRR (1-point on RPE) or 5-min duration as tolerated over sessions to eventually reach the target dose of 65–75% of HRR or RPE 12–14 for 50 mins a session. The adjustment period lasted for an average of 6–8 weeks. Each session also included a 5-min cardiac warm-up and a 5-min cardiac cool-down before and after the prescribed cycling dose for the session. The control exercise was seated low-intensity stretching at <20% of HRR and RPE 9 with its frequency and session duration matched to those of cycling. The total duration of the exercise programs, including the adjustment period, was 6 months (72 total sessions). A Master's-prepared interventionist supervised every session at no more than 1:3 interventionist-to-participants ratio and monitored heart rate and RPE every 5 mins, blood pressure every 10–15 mins, and overexertion signs and symptoms (Yu et al., 2014).

### Outcomes

The outcomes included hippocampal volume, temporal meta-ROI cortical thickness, WMH volume from structural MRI, and NFQ from the resting state functional MRI (rs-fMRI). The protocol for the anatomic sequences and the study data extracted from each sequence were as follows: magnetization prepared rapid gradient echo imaging (MPRAGE) for measuring ROI-wise brain volumes and cortical thickness, axial T2 star for assessing cerebral micro hemorrhages, T2 fluid-attenuated inversion recovery (FLAIR) for assessing cerebrovascular disease, axial diffusion-weighted imaging (DWI) for assessing acute hemorrhage, and axial multiband fMRI for assessing regional brain perfusion.

**Figure 1:**
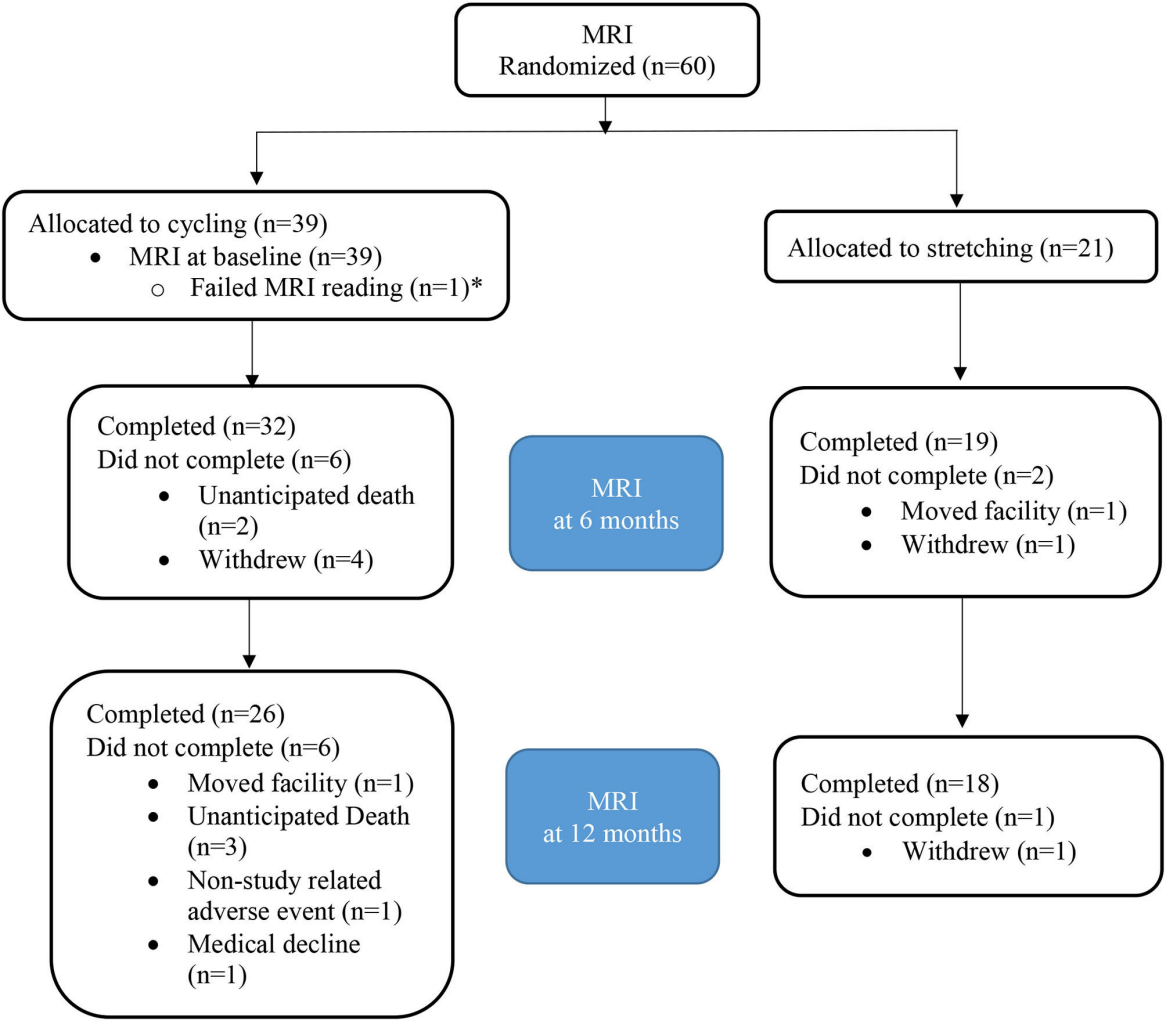
CONSORT diagram for MRI. ^*^Patient with failed MRI at baseline excluded as comparison to baseline not available.

Hippocampal volume and cortical thickness of temporal meta-ROI (entorhinal cortex, fusiform, inferior temporal, and middle temporal gyri) (Jack et al., 2017) as shown in Figure 1 were determined using FreeSurfer (v5.3) (Fischl and Dale, 2000; Fischl et al., 2002). Volume/thickness values were generated for 122 ROIs for each scan. ROI values for hippocampal volume from right and left hemispheres were combined. Global cerebral WMH volume was measured from FLAIR images using a semiautomated segmentation algorithm developed at ADIR (Graff-Radford et al., 2019).

NFQ was calculated from rs-fMRI, following the method described in Wiepert et al. (2017). rs-fMRI data were preprocessed by applying slice timing correction, de-spiking, intra-series motion correction, and simultaneous band pass filtering and nuisance regression. Prior to band pass and nuisance regression steps, a mean-over-time fMRI (3D) volume is created and co-registered with a T1-weighted (T1w) MRI for the same subject. Each T1w image has been processed to create tissue probability maps *via* SPM12 unified segmentation. Additionally, atlases included in the Mayo Clinic Adult Lifespan Template are warped into the space of each T1w image using ANTs registration. Using the EPI-to-T1 registration parameters, atlas parcellations and tissue probability estimates were propagated into space of the fMRI series. Nuisance covariates included intra-series motion parameters and first derivatives thereof as well as time series of mean signal in white matter, CSF, and a global signal region. Estimates of local co-activation within functional atlas regions and correlation between regions are combined to calculate the NFQ.

Cognition was measured with the AD Assessment Scale-Cognition (ADAS-Cog). The ADAS-Cog is the most widely used measure of global cognition in AD drug RCTs and assesses orientation, memory, recall, language, and praxis. Its total score is 0–70 with higher scores indicating worse cognitive function. The interrater reliability of ADAS-Cog was 0.65–0.99 and test-retest reliability was 0.51–1.0 (Rosen et al., 1984).

Covariates included demographics (age, sex, and education) collected from interviews. Dementia stage was determined as mild stage if MMSE was **≥**18 and moderate stage if MMSE <18. Exercise adherence was calculated as the percent of attended sessions and the percent of attended sessions that met session intensity and duration goals. Per-protocol adherence was defined as attending >70% sessions and >70% attended sessions met session intensity and duration prescription.

### Statistical Analyses

Baseline demographics and characteristics were summarized using standard descriptive statistics. Continuous variables were tested between groups using *t*-tests and categorical variables were tested using χ^2^ unless expected cell counts were small, in which case Fischer exact tests were used. Changes at 6 months and at 12 months from baseline were tested using one-way ANCOVA within each group and between groups, following intention-to-treat. In addition, pairwise correlation analyses were conducted to examine the associations of the longitudinal changes in the MRI biomarkers and cognition over 6 and 12 months. Statistical analysis was completed using SAS version 9.4 and *p* < 0.05 was considered statistically significant.

## Results

**Figure 2:**
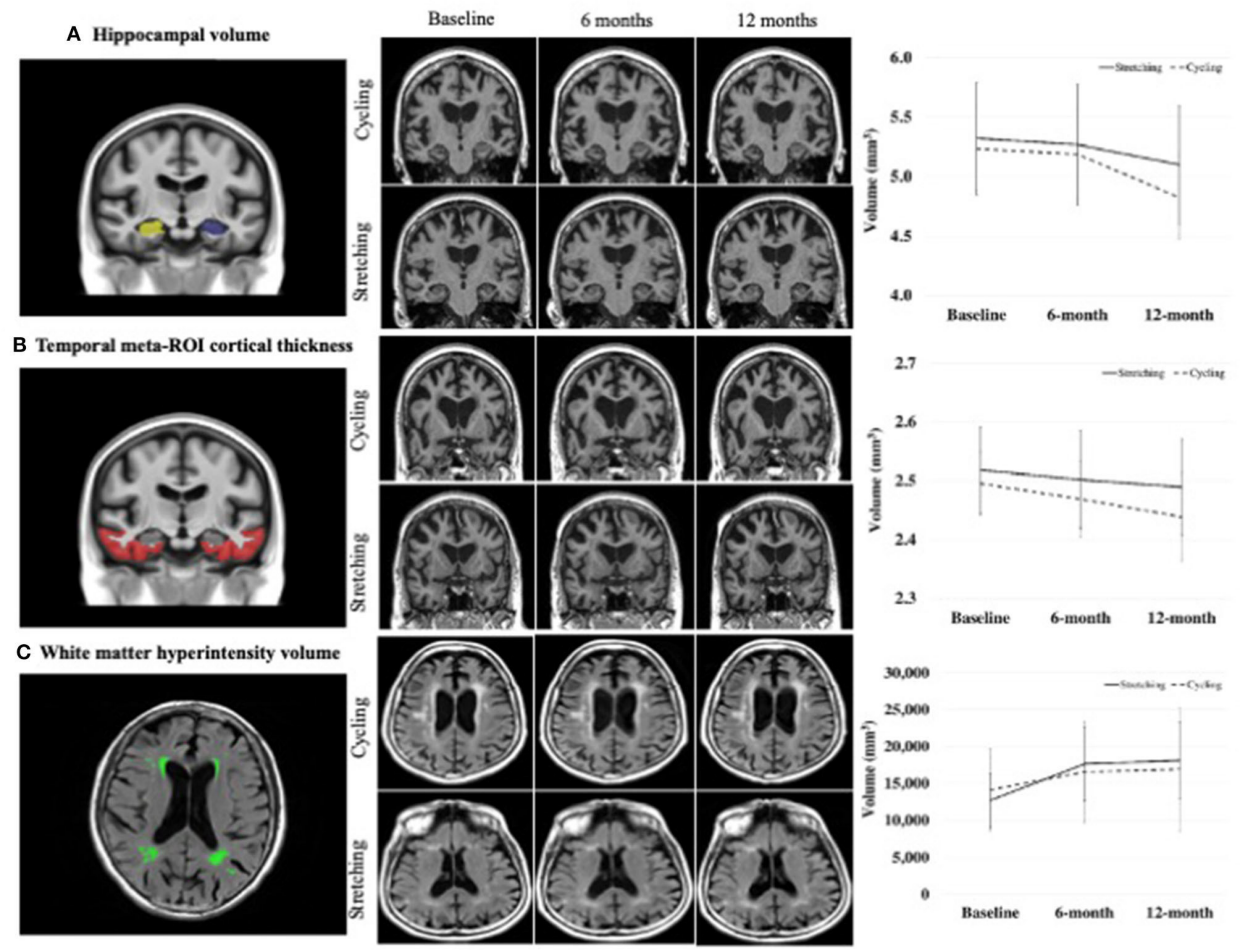
Comparison of changes in MRI biomarkers between groups.

Sixty participants were qualified for the MRI component. One MRI scan at baseline did not meet quality control and was excluded. Among the 59 enrolled participants, 38 were randomized to the intervention group and 21 to the control group (Figure 2). The attrition rate was 13.5% at 6 months (15.8 vs. 9.5% for the intervention vs. control group) and 23.7% at 12 months (28.9 vs. 14.3% for the intervention vs. control group; Table 1), respectively.

**Table 1:** Characteristics of the study MRI sample at baseline (*n* = 59).

	**Overall (*n* = 59) Mean (SD) or *n* (%)**	**Cycling (*n* = 38) Mean (SD) or *n* (%)**	**Stretching (*n* = 21) Mean (SD) or *n* (%)**	*t* or χ^2^^*^	***p***
**Demographics**					
Age, years	77.3 (6.3)	77.2 (6.3)	77.4 (6.5)	0.16	0.876
Sex				1.22	0.268
Male	31 (53%)	22 (58%)	9 (43%)		
Female	28 (47%)	16 (42%)	12 (57%)		
Race/Ethnicity				FET	0.041
Non-Hispanic White	56 (95%)	38 (100%)	18 (86%)		
Hispanic White	2 (3%)	0	2 (10%)		
Black or African American	1 (2%)	0	1 (5%)		
Education, years completed	15.6 (2.6)	15.5 (2.4)	15.8 (3)	0.42	0.673
Marital status				FET	0.685
Married	41 (69%)	27 (71%)	14 (67%)		
Divorced	6 (10%)	4 (11%)	2 (10%)		
Widowed	11 (19%)	7 (18%)	4 (19%)		
Live as married	1 (2%)	0 (0%)	1 (5%)		
Never married	0 (0%)	0 (0%)	0 (0%)		
Living arrangement				FET	0.740
Alone	12 (20%)	8 (21%)	4 (19%)		
With spouse/partner only	35 (59%)	23 (61%)	12 (57%)		
With spouse/partner and other	5 (8%)	2 (5%)	3 (14%)		
With family	7 (12%)	5 (13%)	2 (10%)		
Other	0 (0%)	0 (0%)	0 (0%)		
**Clinical indicators**					
Dementia severity (MMSE)	21.7 (3.2)	21.5 (3.5)	22.2 (2.7)	0.87	0.390
Body Mass Index (BMI)	27.4 (4.7)	27.2 (4.4)	27.7 (5.3)	0.34	0.736
AD medication				0.44	0.508
Any AD medication	36 (61%)	22 (58%)	14 (67%)		
Not used	23 (39%)	16 (42%)	7 (33%)		
# comorbidities	6 (1.9)	6.3 (1.7)	5.5 (2)	−1.63	0.110
NPI-Q symptom presence	3.4 (2.1)	3.8 (2.0)	2.8 (2.2)	−1.67	0.100
NPI-Q severity	5.1 (3.8)	5.7 (3.7)	4.0 (3.7)	−1.66	0.102
NPI-Q caregiver distress	7.1 (5.7)	7.7 (5.0)	6.0 (6.9)	−1.10	0.274
ADL (DAD percent score)	76.9 (16.3)	76.7 (13.0)	77.3 (21.2)	0.12	0.906
Premorbid intellect (WTAR)	35.2 (10.7)	35.9 (9.4)	33.9 (12.8)	−0.72	0.476
**Attrition**					
6-month attrition	8 (13.5%)	6 (15.8%)	2 (9.5%)	FET	0.257
12-month attrition	14 (23.7%)	11 (28.9%)	3 (14.3%)	FET	0.338
**Exercise adherence**					
Sessions attended	55.8 (20.3)	55.5 (19.4)	56.2 (22.4)	0.12	0.906
Attended session meeting prescription	75 (23)	70 (23)	88 (19)	3.06	0.003
Per protocol	32 (54%)	18 (47%)	14 (67%)	2.03	0.154
**MRI measures**					
Hippocampal volume	5.266 (1.090)	5.236 (1.099)	5.320 (1.099)	0.08	0.780
AD-signature cortical thickness	2.504 (0.170)	2.495 (0.170)	2.518 (0.172)	0.24	0.629
WMH volume	13,608 (14,710)	14,122 (17,321)	12,679 (8,432)	0.13	0.722
NFQ	0.525 (0.221)	0.550 (0.204)	0.480 (0.247)	1.36	0.249

* *FET, Fisher Exact Test, if expected cell counts ≤ 5; AD, Alzheimer's disease; DAD, disability in Alzheimer's Disease; MMSE, mini-mental state examination; NPI-Q, neuropsychiatric inventory-caregiver; WTAR, Wechsler Test of Adult Reading*.

Overall, the participants were 77.3±6.3 years old and had 15.6±2.9 years of education with 53% male and 95% non-Hispanic white. The two groups did not differ except that the intervention groups were all white (Table 1).

The 6- and 12-month within-group changes in hippocampal volume, temporal meta-ROI cortical thickness, and WMH volume were statistically significant for both the cycling and stretching groups, but not in NFQ (Table 2). From baseline to 6 months, hippocampal volumes decreased 2.64% in the cycling group vs. 2.89% in the stretching group, temporal meta-ROI cortical thickness decreased 0.94% in cycling and 1.54% in stretching, and WMH volume increased 10.94% in cycling and 24.54% in stretching. From baseline to 12 months, hippocampal volumes decreased 4.47% in the cycling group vs. 3.84% in the stretching group, temporal meta-ROI cortical thickness decreased 2.27% in cycling and 1.79% in stretching, and WMH volume increased 12.08% in cycling and 27.59% in stretching.

**Table 2:** Adjusted 6- and 12-month Changes in Imaging Biomarkers.

**Measure**	**Cycling**		**Stretching**		**Between-group** **difference**
	**Mean (SD)**	***p*** **(from baseline)**	**Mean (SD)**	***p*** **(from baseline)**	***p***
**6-month changes**					
Hippocampal volume	−0.139 (0.198)	0.001^*^	−0.155 (0.142)	0.001^*^	0.759
Temporal meta–ROI cortical thickness	−0.024 (0.040)	0.002^*^	−0.039 (0.047)	0.001^*^	0.156
WMH volume	1534 (2270)	0.001^*^	3381 (3612)	0.001^*^	0.030^*^
NFQ	0.029 (0.258)	0.531	0.038 (0.247)	0.517	0.687
**12-month changes**					
Hippocampal volume	−0.227 (0.185)	0.001^*^	−0.201 (0.142)	0.001^*^	0.886
Temporal meta-ROI cortical thickness	−0.057 (0.057)	0.001^*^	−0.045 (0.053)	0.002^*^	0.936
WMH volume	1829 (2185)	0.001^*^	3764 (3348)	0.001^*^	0.015^*^
NFQ	−0.057 (0.266)	0.285	0.079 (0.357)	0.377	0.174

* *p < 0.05*.

The 6-month and 12-month between-group changes in hippocampal volume, temporal meta-ROI cortical thickness, and NFQ were not statistically significant. The 6- and 12-month changes in WMH volume were statistically significant, favoring the cycling group (Table 2).

The results of pairwise correlation analyses showed a significant negative correlation between 6-month changes in hippocampal volume and ADAS-Cog (*r* = −0.35, *p* < 0.05). The 12-month changes in hippocampal volume and ADAS-Cog were not significant (Table 3). The 6- and 12-month changes in hippocampal volume and temporal meta-ROI cortical thickness were significant (*r* = 0.38 and 0.45 respectively, *p* < 0.01) and 6-month changes in cortical thickness and WMH volume (*r* = −0.37, *p* < 0.01). However, the 6- and 12-month changes in temporal meta-ROI, WMH volume, and NFQ were not significantly associated with the 6- and 12-month changes in ADAS-Cog (Table 3).

**Table 3:** Pairwise correlation coefficients between changes in MRI biomarkers and changes in cognition over 6 and 12 months.

	**(1)**	**(2)**	**(3)**	**(4)**	**(5)**
**6-month changes in:**					
(1) ADAS-Cog	1.00				
(2) Hippocampal volume	−0.34*	1.00			
(3) Cortical thickness	−0.15	0.38**	1.00		
(4) WMH volume	−0.22	−0.09	−0.37**	1.00	
(5) Network failure quotient	0.09	0.06	0.03	−0.11	1.00
**12-month changes in:**					
(1) ADAS-Cog	1.00				
(2) Hippocampal volume	−0.21	1.00			
(3) Cortical thickness	−0.21	0.45**	1.00		
(4) WMH volume	−0.07	−0.03	−0.19	1.00	
(5) Network failure quotient	0.19	0.04	−0.13	0.14	1.00

*p < 0.05; **p < 0.01; two tailed; ADAS-Cog, Alzheimer's disease assessment scale-cognition.

## Discussions

The disease-modifying potential of aerobic exercise for AD has been well-supported in animal research, showing that exercise increases BDNF and reduces AD amyloid plaques and neurofibrillary tangles (McGurran et al., 2019; da Costa Daniele et al., 2020). However, mechanistic studies of effects of aerobic exercise in humans are limited, particularly in older adults with AD dementia, using MRI. An important feature of this study was that the diagnosis of AD dementia was based on clinical criteria and was not verified by biomarkers. Therefore, it is possible that AD played minimal to no role in the cognitive presentation of some participants. Our findings show that hippocampal volume and temporal meta-ROI cortical thickness decreased over 6 and 12 months. The decreases were smaller in the cycling group than the stretching group over 6 months (2.64 vs. 2.89% for hippocampal volume; 0.94 vs. 1.54% for temporal meta-ROI cortical thickness), but greater over 12 months (4.47 vs. 3.84% for hippocampal volume; 2.27 vs. 1.79% for temporal meta-ROI cortical thickness). The between-group differences did not reach statistical significance. We further found that NFQ did not differ within- or between-groups over 6 and 12 months. In contrast, there are significant within- and between-group differences in WMH volume with the cycling group experiencing substantial less WMH volume increases over both 6 and 12 months. There was a significant negative correlation between 6-month changes in hippocampal volume and ADAS-Cog.

Our findings on hippocampal volume and temporal meta-ROI cortical thickness are consistent with the literature. An important reason for our findings and similar findings that failed to show significant effects of aerobic exercise on these variables is the small detectable changes (Villemagne et al., 2013). For example, the mean annualized rate of hippocampal atrophy was 3.5–3.98% or 1.75–1.99% over 6 months in older adults with AD dementia (Jack et al., 2000). Our participants showed greater 6-month decreases in hippocampal volume (2.64% in the cycling group and 2.89% in the stretching group) than expected (Jack et al., 2000; Morris et al., 2017). In a recent study, aerobic exercise was reported to likely attenuate hippocampal volume decline (0.8% in the intervention group vs. 1.6% in the control group) (Morris et al., 2017). While our cycling group also showed a smaller decline, the between-group difference is smaller (0.23%) than that (0.8%) reported in the other trial (Morris et al., 2017). Similar small detectable changes in PET-quantified brain Aβ have also been reported (Villemagne et al., 2013). In addition, we found a significant negative correlation between 6-month changes in hippocampal volume and ADAS-Cog, suggesting that hippocampal atrophy was significantly associated with cognitive decline during the intervention period. This finding further supports hippocampal atrophy as a potential biological mechanism mediating intervention effects. Together, these results explain the inconsistent and negative results from RCTs because these RCTs including ours are usually not powered to detect such small changes in biomarkers. Future RCTs with large sample sizes and powered on biomarkers such as hippocampal volume are needed to truly establish the disease-modifying abilities of aerobic exercise in AD dementia.

Our participants showed that their temporal meta-ROI cortical thickness decreased 0.94% in cycling and 1.54% for the stretching group over the 6-month intervention period, but the between-group differences were not significant. Two recent trials reported that aerobic exercise alone or in combination with cognitive training increased temporal meta-ROI cortical thickness in older adults with preclinical AD (Bae et al., 2020; Um et al., 2020). Our findings further show that over 12 months, temporal meta-ROI cortical thickness decreased 2.27% in the cycling group and 1.79% in the stretching group. These findings indicate that the effects of aerobic exercise on hippocampal volumes and temporal meta-ROI cortical thickness are most likely intervention dependent. Once the intervention stops, the benefits disappear, and the rate of atrophy appears to accelerate in the intervention group. They may also support the hypotheses that the rates of decline in hippocampal volumes and temporal meta-ROI cortical thickness are likely not linear and are heterogeneous as those who declined faster clinically experienced greater decline in hippocampal atrophy (Jack et al., 2000).

Previously, we have reported that our intervention participants had a smaller increase in global cognition as measured by the ADAS-Cog at 6 months (1.0 ± 4.6) than its natural course (3.2 ± 6.3-point increase; *p* = 0.001) (Yu et al., 2021). In this study, we found a significant negative correlation between the 6-month declines in hippocampal volume and the 6-month increases in ADAS-Cog, meaning that hippocampal atrophy was significantly associated with cognitive decline during the intervention period. The 6- and 12-month changes in temporal meta-ROI cortical thickness were significantly associated with the 6- and 12-month changes in hippocampal volume, and 6-month changes in temporal meta-ROI cortical thickness and WMH volume were associated; however, temporal meta-ROI cortical thickness and WMH volume were not associated with ADAS-Cog changes. Together, these findings suggest hippocampal atrophy as a potential biological mechanism mediating intervention effects and aerobic exercise may affect MRI biomarkers differently. Those hypotheses need to be tested in future studies.

Another potential mechanism may play a bigger role in the effect of aerobic exercise: WMH. WMH results from chronic ischemia and is slowly progressive in nature. Aerobic exercise has been postulated as a potential intervention for mitigating WMH progression. Our findings support this postulation and indicate that there are significant within- and between-group differences over both 6 and 12 months with the cycling group experiencing substantial less increases (~50%) of WMH volume than the stretching group. However, a recent trial in vascular dementia did not find significant between-group differences in WMH volume; instead, a sex effect was identified, showing women in the control group experienced more WMH progression than women in the aerobic exercise group (Dao et al., 2019). Future studies are needed to examine the effects of aerobic exercise on WMH and the moderating/mediating factors.

We did not find any significant within- or between-group differences in NFQ over 6 and 12 months, although NFQ is a sensitive measure for functional connectivity (Wiepert et al., 2017). Previously, functional connectivity has been associated with cognition and multimodal exercise intervention was shown to improve functional connectivity in older adults (Li et al., 2014). Functional connectivity may also mediate the association between aerobic fitness and cognition (Voss et al., 2010). A recent study showed that multimodal exercise improved task-based functional connectivity (Boa Sorte Silva et al., 2020). Collectively, these findings suggest the need for large-scale studies and the use of not only rs-fMRI but also task-based MRI for measuring functional connectivity.

The striking differences in mechanistic findings between animal and human studies and the inconsistent findings in human research can be explained by many methodological factors, but the aerobic exercise dose likely plays a major role. Animal studies commonly employed high “duration of exercise to lifespan,” which means high and desired doses were used and achieved (Brown et al., 2019), while human studies are often limited in doses. Inter-individual differences in humans further affect the severity and progression of degenerative pathology and exercise dose delivery and may have hidden a wide variety of biomarker responses to aerobic exercise when the conventional “between-group difference” analysis was performed (Williamson et al., 2017). Heterogenous aerobic fitness responses to aerobic exercise that have long been recognized in young adults (Lortie et al., 1984; Bouchard and Rankinen, 2001; Hautala et al., 2003; Rose and Parfitt, 2007; Karavirta et al., 2011; Robinson et al., 2017; Hecksteden et al., 2018; Ross et al., 2019) were shown to be more prominent in older adults (Sisson et al., 2009) and demonstrated by our FIT-AD Trial for the first time in older adults with AD dementia (Yu et al., 2020). Moreover, effects of exercise might be obscured by the high nonresponse rates. Compared to the 17–19% aerobic fitness nonresponse rates to aerobic exercise in young adults (Higgins et al., 2015; Gurd et al., 2016), the nonresponse rates were 19.3–44.9% in older adults when nonresponse was defined as no improvement (Sisson et al., 2009) and rose to 63.4% when nonresponse was defined as <5% improvement (Pandey et al., 2015).

The strengths of our study include a rigorous design and implementation following trial guidelines, the objective measurement of delivered exercise doses, high adherence, the match of exercise frequency and duration between groups, and the use of validated outcome measures.

Weaknesses that inform future trial design include the lack of power to detect between-group differences, the inclusion of both mild and moderate stages of AD dementia, and the unequal representation of mild and moderate stages of AD dementia. In addition, environments might have influenced intervention delivery and adherence. Both the YMCA gym and the lounge of the senior community had open floor plan. Although we delivered exercises during the down time at the YMCA gym, the gym was more crowded and nosier than the lounge of the senior community. Last, our study could have been strengthened with a usual care control group to observe the natural course of changes since both exercises appeared to affect the outcomes. The decision of not including usual care was not made lightly and was influenced by multiple factors (e.g., considerations of the effects of attention and social interaction, the feasibility of recruitment, and retention).

To conclude, aerobic exercise may reduce the decline in hippocampal volume and temporal meta-ROI cortical thickness, but the effect sizes are likely to be small and dose-dependent and reverse once intervention stops. Aerobic exercise appears to slow down WMH progression. Hippocampal atrophy was associated with cognitive decline during the intervention period.

## Data Availability Statement

The raw data supporting the conclusions of this article will be made available by the authors, without undue reservation.

## Ethics Statement

The studies involving human participants were reviewed and approved by the University of Minnesota's Institutional Review Board. The patients/participants and their surrogates provided their written informed consent to participate in this study.

## Author Contributions

FY designed and supervised all aspects of the study implementation and drafted the manuscript. CJ designed all aspects of the study related to the MRI, supervised MRI data collections, and contributed to the draft of the manuscript. MM and SH conducted data analyses, developed figures and tables, and contributed to the draft of the manuscript. JG, DJ, and HB analyzed MRI data and contributed to the draft of the manuscript. All authors read and approved the final manuscript.

## Funding

The research study reported in this publication was supported by the National Institute on Aging of the National Institutes of Health (1R01AG043392-01A1). The CTSI and Center for Magnetic Resonance Resources were supported by the National Institutes of Health National Center for Advancing Translational Sciences of the National Institutes of Health Award Number UL1TR000114 and the National Institute of Biomedical Imaging and Bioengineering Award Number P41 EB1058941, respectively.

## Author Disclaimer

The content is solely the responsibility of the authors and does not necessarily represent the official views of the National Institutes of Health.

## Conflict of Interest

The authors declare that the research was conducted in the absence of any commercial or financial relationships that could be construed as a potential conflict of interest.

## Publisher's Note

All claims expressed in this article are solely those of the authors and do not necessarily represent those of their affiliated organizations, or those of the publisher, the editors and the reviewers. Any product that may be evaluated in this article, or claim that may be made by its manufacturer, is not guaranteed or endorsed by the publisher.
